# Quantitative Radiographic Progression of Joint Space Narrowing in Medial and Lateral Compartment Knee Osteoarthritis After Intraarticular Platelet-Rich Plasma Injection

**DOI:** 10.7759/cureus.88827

**Published:** 2025-07-26

**Authors:** Tyler Schmitz, Jeffrey Nadwodny, Hillary W Garner, Renata Skov, Ahmad Al-Awadi, George G. A Pujalte

**Affiliations:** 1 Sports Medicine, Ascension Medical Group Sacred Heart, Pensacola, USA; 2 Family Medicine and Sports Medicine, Mayo Clinic, Jacksonville, USA; 3 Radiology, Mayo Clinic, Jacksonville, USA; 4 Family Medicine, Mayo Clinic, Jacksonville, USA; 5 Family Medicine, Milton District Hospital, Milton, CAN; 6 Family Medicine, Orthopedics, and Sports Medicine, Mayo Clinic, Jacksonville, USA

**Keywords:** intraarticular injection, kellgren-lawrence, knee osteoarthritis, platelet-rich plasma, quantitative, radiography

## Abstract

Introduction: Knee osteoarthritis (KOA) is a common pathology of the knee. Intraarticular injection of platelet-rich plasma (PRP) can be used as a nonoperative therapy to improve symptoms. The impact of PRP on structural derangements of KOA has not been widely studied. Although radiography is an appropriate imaging modality for initial evaluation and follow-up, many published studies evaluating for structural change in KOA use magnetic resonance imaging or ultrasound.

Objective: The aim of our study was to evaluate for change in osteoarthritis severity and joint space width (JSW) on radiography in patients with KOA treated with PRP.

Methods: Included study patients had pre-injection and post-injection posterior-anterior flexion radiography obtained. Severity of osteoarthritis was assessed using the Kellgren-Lawrence grade, and JSW was measured.

Results: Sixteen patients with 20 PRP-treated knees (13 medial-compartment-predominant and seven lateral-compartment-predominant KOA) were included in the analysis. Severity grade did not change in 18 of the 20 knees but worsened in two knees at follow-up. A median change of −0.1 mm in JSW after PRP injection at a median follow-up of 11.5 months was seen in both lateral- and medial-compartment-predominant knees, which is less than the expected annual rate of joint space narrowing in untreated KOA.

Conclusion: Our study was underpowered and therefore was not able to demonstrate a significant structural change following PRP treatment using a radiographic comparison model. Larger studies are needed to provide further assessment on the structural impact of PRP injection in KOA.

## Introduction

Knee osteoarthritis (KOA) is a progressive disease that involves the tibiofemoral and patellofemoral joints and is the most common single cause of lower-limb disability in adults older than 50 years [1]. On a global scale, KOA affects approximately 654.1 million people over the age of 40 worldwide [2]. The most common symptoms of KOA are joint pain, stiffness, and restriction in locomotion [1].

Although conservative therapies, such as weight loss, physical therapy, and corticosteroid joint injections, are often trialed, many patients ultimately undergo knee arthroplasty, which is responsible for over $11 billion in healthcare costs in the United States per year [3]. Given the current high prevalence of KOA and its predicted progressive rise in the coming years due to population aging, there has been increased focus on therapeutic options other than surgery. One therapy alternative that has shown promise is the intraarticular injection of platelet-rich plasma (PRP).

In vivo and in vitro experiments of PRP treatment have shown favorable gene expression leading to decreased proinflammatory mediators, reduced protease activity and production, decreased synovial inflammation, stimulation of articular cartilage and synovium cell proliferation, promotion of extracellular matrix repair, and slowing of cartilage degeneration [4-11].

Radiography is the most widely used imaging modality for the diagnosis of KOA and allows for the detection of characteristic features of KOA, including marginal osteophytes, joint space narrowing (JSN), subchondral sclerosis, and subchondral cysts [12,13]. Minimum joint space width (JSW) measured on a weight-bearing posterior-anterior (PA) radiograph of the knee has been shown to be the most prevalent, reproducible, and sensitive imaging finding for KOA [14-17]. The sensitivity for KOA changes has been reported to be 75% for weight-bearing radiography vs 70.8% for magnetic resonance imaging (MRI) [18].

Several small studies evaluating MRI findings in KOA after PRP injection have demonstrated mixed results. Some have reported improvement in patellofemoral cartilage volume, synovitis, meniscal integrity, and KOA grade [19,20], while others have shown no significant structural changes [21-23]. However, despite growing interest in PRP, objective radiographic assessment of structural outcomes remains underexplored. For example, although Buendía-López et al. conducted a randomized trial with 52-week follow-up radiographs comparing PRP, hyaluronic acid (HA), and non-steroidal anti-inflammatory drugs (NSAIDs), the study relied on Kellgren-Lawrence (KL) grading without quantitative joint space assessment [21]. To date, most literature emphasizes symptom relief, MRI findings, or qualitative radiographic scores, with little focus on minimum JSW, a validated and reproducible metric for monitoring cartilage loss in KOA [17]. Furthermore, inconsistencies in PRP composition, injection timing, and imaging intervals further cloud the interpretation of PRP's structural effects [24]. Given that radiographic minimum JSW is a clinically relevant marker and practical in routine settings, our study addresses this gap by using weight-bearing PA flexion radiography to evaluate changes in both JSW and KL grade following PRP treatment in medial and lateral compartment KOA.

Data from this study was presented at the American Medical Society for Sports Medicine Annual Meeting in Austin, Texas, April 8-13, 2022. The abstract for this presentation has been published: Schmitz T, Pujalte G, Garner H, Al-Awadi A, Nadwodny J. Quantitative Radiographic Progression of Joint Space Narrowing in Knee Osteoarthritis After Platelet Rich Plasma Injection. Clin J Sport Med 2022; 32(2):218.

## Materials and methods

This retrospective observational study was reviewed by expedited review procedures and is determined to be exempt from the requirement of IRB approval by the Mayo Clinic Institutional Review Board (application number: 20-009105). Due to the retrospective nature of the study and minimal risk to participants, the requirement for informed consent was waived.

Clinical and imaging records of patients aged 18 years or older who had been diagnosed with medial or lateral compartment KOA and had received at least one intraarticular knee PRP injection over a one-year period were retrospectively reviewed. An initial 151 patients who received a PRP knee injection were identified. After applying the inclusion and exclusion criteria, a total of 20 knees from 16 patients were included in the final analysis. Most were excluded due to not having follow-up radiography, having an alternative injection within the radiographic window, receiving a stem cell injection, or having an exclusionary diagnosis.

Patients were excluded if they had a diagnosis of rheumatoid arthritis, chronic septic arthritis, inflammatory arthritis, or post-traumatic arthritis or if they received any intraarticular injection other than PRP in the same knee within three months prior to or at any point after the PRP injection. Patients without pre- and post-injection standardized, nonmagnified, full weight-bearing PA knee radiographs in 20-30° flexion (Lyon Schuss view) were also excluded. Additionally, each PA flexion radiograph was evaluated for appropriate alignment, defined as ≤1 mm distance between the anterior and posterior margins of the medial tibial plateau on the PA view [15-17]. Knees with malaligned or rotated radiographs were excluded to ensure the accurate measurement of minimum JSW.

PRP preparation and injection procedures followed the standard clinical protocol used at the Mayo Clinic in Florida, the institution where the study was conducted. All patients received leukocyte-poor PRP, which was prepared using a standardized, commercially available centrifugation system. Peripheral venous blood was drawn into anticoagulant-containing tubes and centrifuged in a two-step process to separate the PRP layer. The final PRP product was extracted under sterile conditions, yielding a solution with concentrated platelets and minimal leukocyte content. Although exact platelet concentrations were not routinely measured in this retrospective review, all PRP preparations were derived from this uniform method.

Injections were performed under sterile conditions using a lateral mid-patellar approach with the patient in a supine position and the knee in slight flexion. A single intraarticular injection of PRP was delivered directly into the knee joint using a 22-gauge needle. The volume of PRP administered ranged from approximately 4 mL to 6 mL, depending on the volume obtained after centrifugation. No ultrasound guidance was used, consistent with standard practice at the institution.

The vertical distance between the cortical edge of the femoral condyle and the cortical edge of the tibial plateau at the site of greatest narrowing was measured on the PA flexion view using digital caliper tools within the imaging software and recorded as the minimum JSW. The compartment with the greatest JSN (either medial or lateral) was selected for each knee and used consistently for pre- and post-injection comparisons.

Minimum JSW measurements were performed by a musculoskeletal radiologist with experience in radiographic KOA assessment and reviewed by a second trained investigator. Reviewers were not blinded to the temporal sequence of the radiographs (pre- or post-injection), as side-by-side image comparison was necessary to ensure consistent alignment and measurement positioning. Given the retrospective nature of the study and small sample size, formal assessment of inter- or intra-rater reliability was not conducted. However, discrepancies in measurement were discussed and resolved by consensus between the two reviewers to improve consistency.

Osteoarthritis severity was graded using the KL classification system (grades 0 to IV), the most widely used radiographic scoring method for KOA [25]. Each radiograph was evaluated by a musculoskeletal radiologist and a second reviewer, with discrepancies resolved by consensus. The grading definitions used in this study are summarized in Table 1, as described in the classification system originally published by KL and adopted in subsequent radiographic KOA studies [14,26].

**Table 1:** Kellgren-Lawrence grading system for osteoarthritis From Audrey et al. [14]; Licensee Bentham Open. This is an open access article and distributed under the terms of the Creative Commons Attribution 4.0 International Public License (CC-BY 4.0) (https://creativecommons.org/licenses/by/4.0/legalcode) which permits unrestricted use, distribution, and reproduction in any medium, provided the original author and source are credited

Grade	Radiological findings
0	No radiological findings of osteoarthritis
I	Doubtful narrowing of joint space and possible osteophytic lipping
II	Definite osteophytes and possible narrowing of joint space
III	Moderate multiple osteophytes, definite narrowing of joint space, small pseudocystic areas with sclerotic walls, and possible deformity of bone contour
IV	Large osteophytes, marked narrowing of joint space, severe sclerosis, and definite deformity of bone contour

For each treated knee, the following data were collected: patient age and sex, laterality (left or right), number of PRP injections received, timing between radiographs and injection(s), JSW, and KL grade before and after injection.

Descriptive statistics were used to summarize baseline characteristics and outcomes. Continuous variables were reported as median with range and categorical variables as frequency and percentage. Changes in JSW between pre- and post-injection radiographs were analyzed descriptively due to the small sample size and non-normal data distribution. No formal inferential testing was applied. Instead, individual-level changes in JSW were illustrated using line plots to visualize trends over time in both medial- and lateral-compartment-dominant knees.

## Results

**Figure 1:**
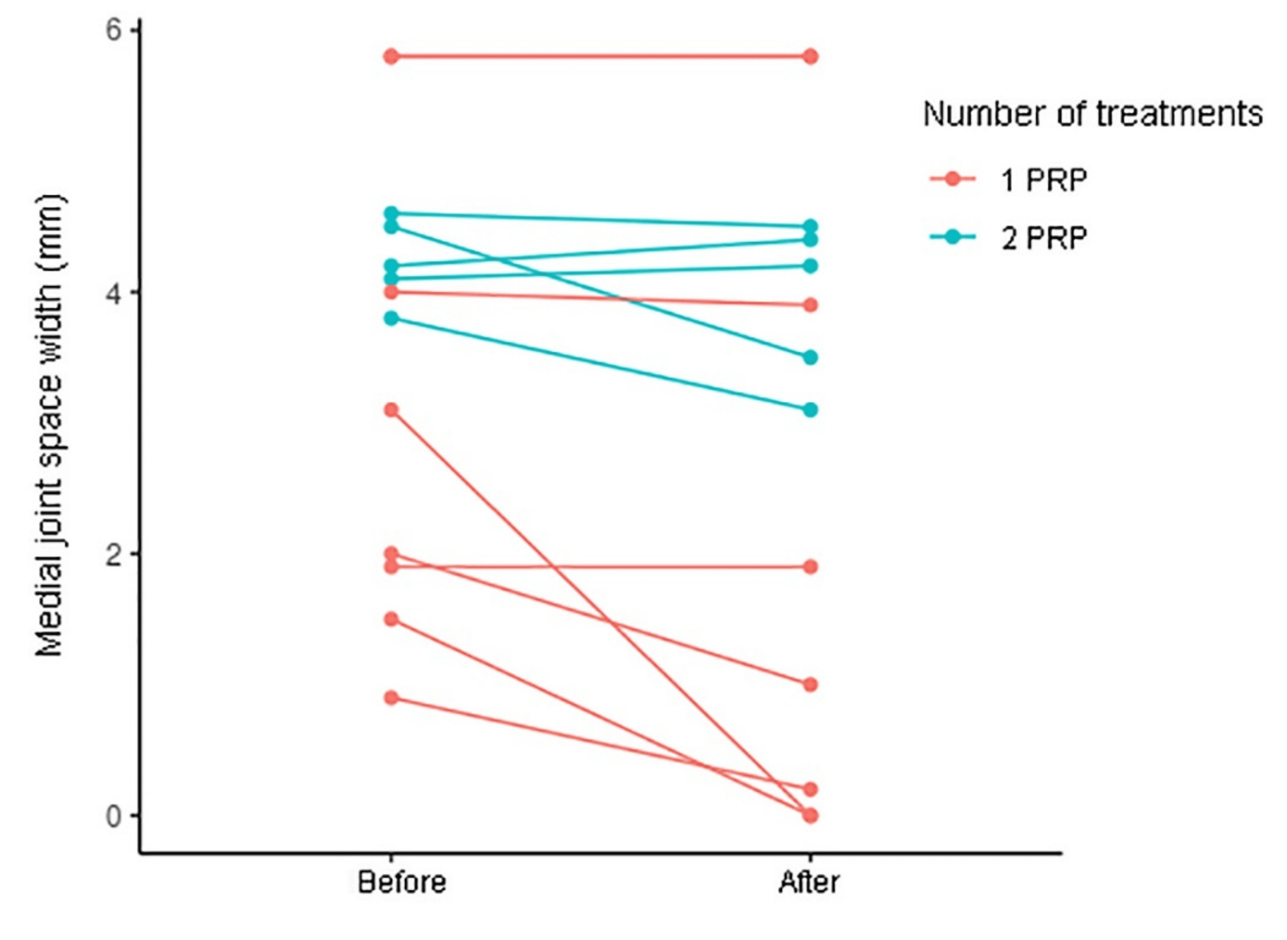
Medial joint space width on posterior-anterior flexion view before and after PRP PRP: platelet-rich plasma

**Figure 2:**
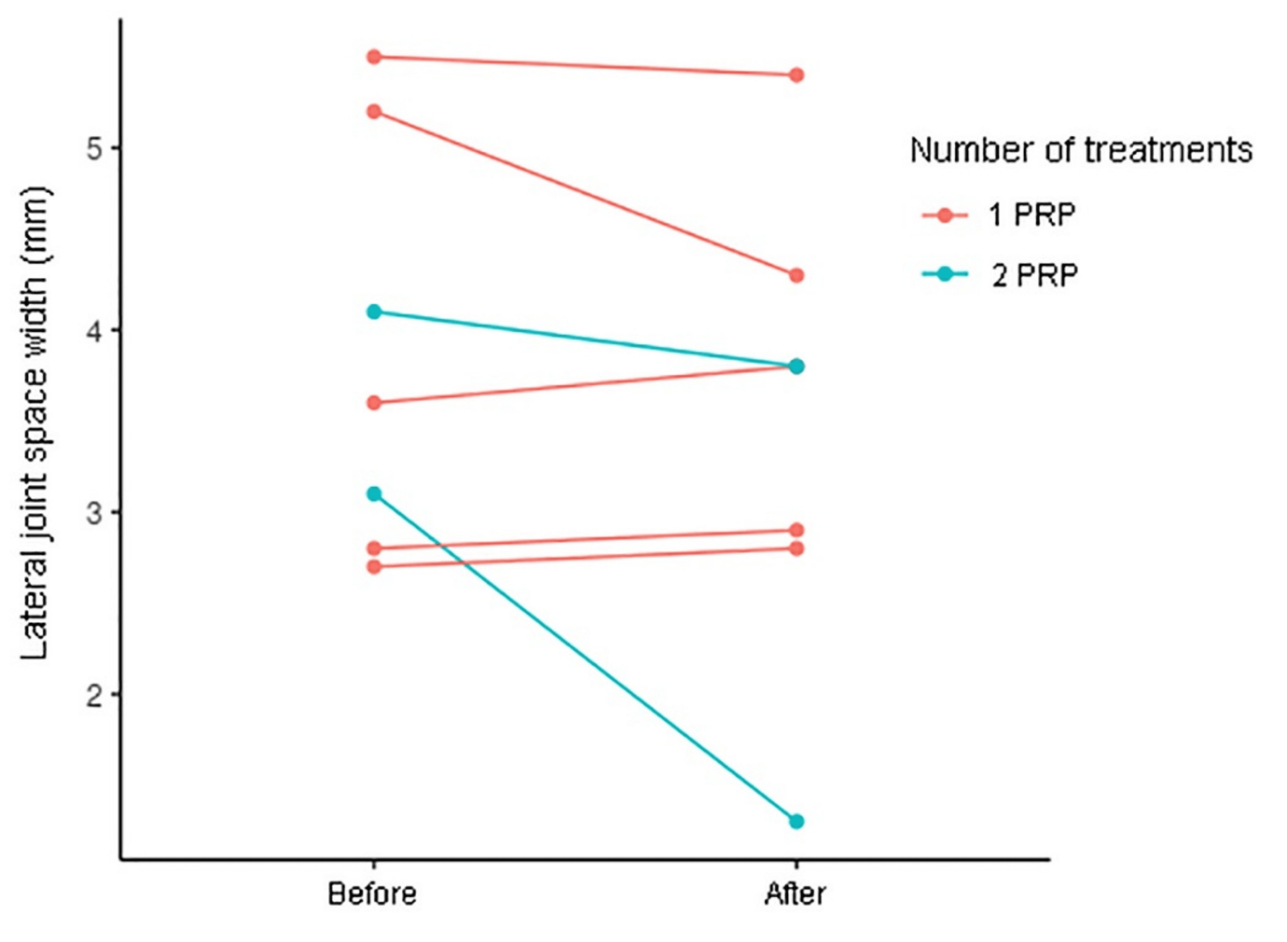
Lateral joint space width on posterior-anterior flexion view before and after PRP PRP: platelet-rich plasma

In total, 20 knees with KOA from 16 patients (nine men (56.3%) and seven women (43.8%)) were included in the study. Patient age at the time of the first PRP injection ranged from 41 to 89, with a mean age of 62.7. The medial compartment was measured in 13 knees (65%), and the lateral compartment was measured in seven (35%). Thirteen knees (65%) were treated with one PRP injection, and seven were treated with two. The median time interval between pre-injection radiography and the first PRP injection was two months, and the median time interval between the PRP injection and post-injection radiography was 11.5 months. There was a median change in JSW of −0.1 mm between pre-injection and post-injection for both medial-compartment-predominant and lateral-compartment-predominant KOA (Figure 1 and Figure 2). Severity scores stayed the same for most knees; however, for two, the severity score increased 1 grade after PRP (Table 2).

**Table 2:** Patient demographics and baseline characteristics based on each surgery JSW: joint space width; KOA: knee osteoarthritis; PA: posterior-anterior; PRP: platelet-rich plasma; mo: months; L: left; R: right

Characteristics	1 PRP (n=13)	2 PRP (n=7)	Total (n=20)
Sex, no. (%)			
Not reported	3	1	4
Female	5 (50.0)	2 (33.3)	7 (43.8)
Male	5 (50.0)	4 (66.7)	9 (56.2)
Knee, no. (%)			
L	4 (30.8)	3 (42.9)	7 (35.0)
R	9 (69.2)	4 (57.1)	13 (65.0)
Pre-PRP radiography timing, first treatment, median (range), mo	1.5 (0.0, 10.0)	3.5 (0.0, 11.0)	2.0 (0.0, 11.0)
Post-PRP radiography timing, first treatment, median (range), mo	9.0 (1.0, 24.0)	15.0 (8.0, 22.0)	11.8 (1.0, 24.0)
Pre-PRP radiography timing, second treatment, median (range), mo	NA	12.0 (4.0, 18.0)	12.0 (4.0, 18.0)
Post-PRP radiography timing, second treatment, median (range), mo	NA	9.0 (1.0, 21.0)	9.0 (1.0, 21.0)
Compartment predominance, no. (%)			
Lateral	5 (38.5)	2 (28.6)	7 (35.0)
Medial	8 (61.5)	5 (71.4)	13 (65.0)
Medial JSW on PA flexion view, median (range), mm	8	5	13
Pre-PRP	2.5 (0.9, 5.8)	4.2 (3.8, 4.6)	4.0 (0.9, 5.8)
Post-PRP	1.4 (0.0, 5.8)	4.2 (3.1, 4.5)	3.5 (0.0, 5.8)
Change	−0.4 (−3.1, 0.0)	−0.1 (−1.0, 0.2)	−0.1 (−3.1, 0.2)
Lateral JSW on PA flexion view, median (range), mm	5	2	7
Pre-PRP	3.6 (2.7, 5.5)	3.6 (3.1, 4.1)	3.6 (2.7, 5.5)
Post-PRP	3.8 (2.8, 5.4)	2.5 (1.3, 3.8)	3.8 (1.3, 5.4)
Change	0.1 (−0.9, 0.2)	−1.0 (−1.8, −0.3)	−0.1 (−1.8, 0.2)
Pre-PRP tricompartmental KOA severity (Kellgren-Lawrence grade), no. (%)			
II	2 (15.4)	1 (14.3)	3 (15.0)
III	3 (23.1)	5 (71.4)	8 (40.0)
IV	8 (61.5)	1 (14.3)	9 (45.0)
Post-PRP tricompartmental KOA severity (Kellgren-Lawrence grade), no. (%)			
II	2 (15.4)	1 (14.3)	3 (15.0)
III	3 (23.1)	3 (42.9)	6 (30.0)
IV	8 (61.5)	3 (42.9)	11 (55.0)
Severity change, median (range)	0.0 (0.0, 0.0)	0.0 (0.0, 0.0)	0.0 (0.0, 0.0)
Severity category, no. (%)			
Increased	0 (0.0)	2 (28.6)	2 (10.0)
Same	13 (100.0)	5 (71.4)	18 (90.0)

## Discussion

Osteoarthritis is the most common joint pathology and is classically described as degenerative change due to loss of cartilage. The pathogenesis of osteoarthritis is more complex than once thought. Proinflammatory mediators, such as cytokines and chemokines, have been found in synovial fluid and appear to activate the production of proteolytic enzymes [27]. These enzymes are thought to cause the degradation of the cartilage extracellular matrix and structural components commonly seen in osteoarthritis [28]. This environment leads to a loss of articular cartilage and subchondral bone reaction and can cause derangements of the synovial membrane, capsule, menisci, ligaments, and periarticular muscle [29].

There are multiple endpoints for assessing response to osteoarthritis treatment, including symptom improvement and positive structural changes identified with imaging. Radiographic evidence of JSN is often used as an indicator of cartilage loss and has been shown to be a robust surrogate to assess response to osteoarthritis treatment [29]. Emrani et al. [30] reported a mean (SD) rate of JSN of 0.13 (0.15) mm/year in KOA. Benichou et al. [31] reported a mean rate of medial JSN of approximately 0.2 mm over one year. Furthermore, a change in JSN of greater than 0.5 mm over 2-3 years has been shown to be a clinically significant predictor of joint replacement [32]. For the radiographic assessment of JSN, minimum JSW, as indicated by the standardized response mean (sensitivity to change on serial radiography), is a more sensitive measure than mean JSW or joint space area [16,17]. Measuring minimum JSW is performed by delineating the bone edges at the narrowest aspect of the joint space and measuring the interbone distance with a software measuring tool [17]. The most accurate and reproducible view for determining minimum JSW is the weight-bearing PA view of the knee in 20-30° flexion (Lyon Schuss view) [15-17]. This view is ideal because it positions the posterior weight-bearing aspect of the femoral condyle, where maximum cartilage destruction occurs in most patients with KOA, in closest proximity to the tibial plateau [15-17]. Additionally, the alignment between the anterior and posterior margins of the medial tibial plateau with the central radiography beam is very important for determining an accurate minimum JSW [15-17]. The general agreement is that if this alignment interval is less than or equal to 1 mm, the quality of the radioanatomic positioning of the knee is satisfactory; otherwise, if the distance is greater than 1 mm, the quality is unsatisfactory [16,17]. If the positioning is satisfactory, this view has been shown to have an intraobserver κ value of 0.98 in the same image (0.76 in a repeat image) and a standardized response mean of 0.51 (0.25 if unsatisfactory alignment) for measuring minimum JSN [17,33,34]. While the medial tibiofemoral compartment is most commonly affected [1], a significant inverse relationship has been shown between the medial and lateral tibiofemoral compartments in both the anterior-posterior and PA flexion radiographic views [15,16].

Nonoperative treatment for KOA includes nonpharmacologic and pharmacologic measures. Common nonpharmacologic options include physical therapy, exercise, weight loss, local heat and cold, knee braces, insoles, walking aids, and psychological interventions [35]. Common pharmacologic treatments include topical capsaicin, topical and oral NSAIDs, acetaminophen, duloxetine, intraarticular corticosteroid injections, glucosamine and chondroitin, various nutritional supplements, opioids, transcutaneous electrical nerve stimulation, acupuncture, intraarticular HA injections, and intraarticular PRP injections [35]. Regarding PRP specifically, multiple studies have demonstrated improvement in subjective clinical symptoms, including pain and function [36-59]. Several of these studies also showed superiority of PRP over HA [37,38,45,47,50,53] or corticosteroid injections [41,45,53]. However, evidence is still limited by potential study bias and variable study methods in PRP dosing, frequency, and timing intervals.

Although studies have shown PRP to be beneficial in treating symptoms of KOA, there is limited and mixed evidence on the structural impacts of PRP in the knee. Three studies have shown positive structural change following treatment with PRP [19,20,60]. One compared baseline and eight-month follow-up MRI in 42 knees treated with either two PRP injections separated by four weeks or exercise therapy alone [20]. This study reported significant improvement in patellofemoral cartilage volume, synovitis, and meniscal structural integrity in the PRP group but only improvement in patellofemoral cartilage volume in the control group [20]. A second study compared baseline and six-month MRI of knees treated with either three PRP injections (n=29) or three HA injections (n=25) given at four-week intervals [19]. The severity of KOA as assessed by MRI improved by at least 1 grade in 14 of 29 knees in the PRP group, while only two of 25 knees improved in the HA group [19]. The third study compared baseline, three-month, and six-month follow-up ultrasound in 89 patients treated with either three PRP injections (n=45) or three HA injections (n=44) given at two-week intervals [60]. The severity of KOA based on the degree of synovial hypertrophy, synovial vascularity, and knee effusion as assessed by ultrasound was reported to be significantly better in the PRP group at both three-month and six-month follow-up [60].

Our study's finding of a median −0.1 mm change in JSW over ~11.5 months aligns with prior evidence suggesting PRP may help slow the structural progression of osteoarthritis, though not reverse it. A meta-analysis of 30 randomized controlled trials found no significant increase in JSW in treated vs control knees at one year, but PRP groups maintained joint space better than controls, despite both experiencing mild narrowing (p>0.05) [61]. Clinically, PRP also confers meaningful symptom relief: a comprehensive review of 30 randomized controlled trials showed PRP outperformed HA in pain and functional outcomes at six and 12 months [61].

Importantly, our reliance on radiographic JSW, rather than MRI or ultrasound, fills a critical gap: less costly, more broadly available, and often the standard imaging in routine clinical follow-up. While MRI studies like those by Halpern et al. have shown selective improvements in cartilage volume and synovitis after PRP, not all outcomes trend in the same direction, and follow-up durations vary [23]. Our use of minimum JSW on weight-bearing Schuss radiographs, a validated and reproducible metric, adds practical, accessible insight into structural outcomes post-PRP.

Furthermore, variability in PRP formulation (e.g., leukocyte-rich vs leukocyte-poor), dosing regimen, patient osteoarthritis severity, and follow-up timing is well-documented in meta-analyses as a major source of heterogeneity [62]. Our study’s observed structural stability occurs despite such uncontrolled variability, supporting the hypothesis that PRP may modestly retard cartilage loss in real-world settings. However, our underpowered sample limits definitive conclusions.

Looking ahead, large prospective randomized controlled trials are still needed to establish causality. Trials like the RESTORE study (large, placebo-controlled, 288 patients) and Di Martino et al. (five-year follow-up comparing PRP vs HA) have produced valuable clinical outcome data, but they lacked quantitative radiographic JSW analysis [63,64]. Integrating standardized imaging protocols in future randomized controlled trials, ideally combining radial assessments across medial/lateral compartments, will be essential to determine whether PRP offers true long-term structural protection.

While our findings suggest a possible slowing of radiographic progression in PRP-treated knees, these results should be interpreted with caution. The retrospective design, absence of a control group, and potential for selection bias substantially limit the ability to draw causal inferences. It is possible that patients who received follow-up imaging were those experiencing persistent or worsening symptoms, which could have skewed the observed trends. Additionally, variation in follow-up timing and lack of standardized symptom or functional outcome measures further restrict the interpretability of our findings. These limitations underscore the need for larger, prospective, controlled trials with standardized imaging and clinical follow-up to more definitively assess the structural impact of PRP in KOA.

Conversely, three other studies showed no structural changes following treatment with PRP [21-23]. One study compared baseline and six-month MRI in 57 patients treated with a single PRP injection and showed no significant change in tibiofemoral or patellofemoral articular cartilage thickness [22]. A second study compared baseline and 12-month MRI in 15 patients with KL grades 0 to II treated with a single PRP injection and demonstrated no change per compartment in at least 73% of cases at one year [23]. The third study compared baseline and 12-month MRI in 98 randomized patients with KOA treated with either a single PRP injection, a single HA injection, or daily oral NSAIDs [21]. The study found no statistically significant difference in KL progression or cartilage thickness on MRI in any group [21]. 

To our knowledge, this is the first study to assess the impact of PRP on structural changes of KOA using radiography. Most knees in our study had medial compartment predominance (n=13), received only one PRP injection (n=14), and had no change in KL grade (n=18). Interestingly, the two knees that showed worsening of the KL grade had received two PRP injections. This could be because the patients who received two injections had more pain and dysfunction and therefore potentially more rapid progression of KOA. At a median radiographic follow-up of 11.5 months in 20 knees, we found a median change in JSW of −0.1 mm for the predominant KOA compartment. This is slightly less than the rate of narrowing reported by Emrani et al. [30] and Benichou et al. [31] in untreated KOA, but our sample size is too small to confidently attribute the difference to PRP. Although further stratification of JSW change by KL grade would be ideal, this was not possible due to the small number of knees in our study. Overall, our study shows no radiographic improvement in JSW or KL grade after PRP injection at a median follow-up of 11.5 months.

This study has several important limitations that restrict the ability to draw causal conclusions properly. First, a small number of patients met our inclusion criteria. Many patients who had received an intraarticular PRP injection did not have follow-up radiography because either it was not ordered or they had a different type of injection in the same knee within the follow-up period, which disqualified them from inclusion. The patients who did have follow-up radiography may potentially have had this because they were experiencing recurrent pain which may have affected their JSN values. Having follow-up radiography for all patients would have strengthened the results and study. A second limitation is that subjective pain and function scores, age, and body mass index were not considered. It is possible that high pain scores, low function scores, increased age, and increased body mass index could be associated with more progressive JSN. Another limitation is that only the narrowest space in the predominant compartment was measured. Measuring the total volume of joint space within all three knee compartments may have been more accurate but would have required more advanced software. Finally, the study institution primarily uses leukocyte-poor PRP for joint injections; we did not account for the specific type of PRP, volume, or platelet concentration in this study.

In addition to these limitations, the retrospective nature of the study, lack of a control group, and potential for selection bias further limit the ability to infer causality. Without a control group, it is not possible to determine whether the observed changes in JSW were due to PRP treatment or part of the natural progression of osteoarthritis. Furthermore, the absence of blinding during JSW measurements may introduce measurement bias, although this was necessary to ensure accurate alignment and comparison of radiographs. These factors highlight the need for larger, prospective, controlled studies with standardized imaging protocols and blinded assessments to more definitively evaluate the structural effects of PRP in KOA.

## Conclusions

The primary symptoms of KOA are joint pain, stiffness, and locomotor restriction, presumably brought about by the degradation of cartilage extracellular matrix and structural components, which often manifest as radiographic changes. Weight-bearing radiography commonly shows JSN, which correlates with disease severity. In our study, radiographic KOA severity remained stable in most PRP-treated knees, and the median change in JSW was −0.1 mm over a median follow-up of 11.5 months. This degree of change is numerically lower than previously reported annual JSN rates in untreated KOA, which range from approximately 0.13 mm to 0.2 mm per year. However, this observation must be interpreted cautiously, as no statistical comparison was made between our data and historical cohorts and the variability in measurement protocols and patient populations limits direct comparisons. While this finding may suggest a possible slowing of structural deterioration, the study design does not support causal conclusions. Nonetheless, the potential for PRP to contribute to structural preservation remains a promising area for future research. Larger, prospective, controlled studies are needed to determine whether PRP meaningfully impacts the structural progression of KOA.
